# Bacterial Profile and Antimicrobial Resistance Patterns of Diabetic Foot Infections in a Major Research Hospital of Turkey

**DOI:** 10.3390/antibiotics13070599

**Published:** 2024-06-27

**Authors:** Belgin Coşkun, Müge Ayhan, Serap Ulusoy, Rahmet Guner

**Affiliations:** Ankara Bilkent City Hospital, 06700 Ankara, Turkey; muge.ayhan@saglik.gov.tr (M.A.); serap.ulusoy2@saglik.gov.tr (S.U.); haticerahmet.guner@saglik.gov.tr (R.G.)

**Keywords:** antibiotic, antibiotic resistance, diabetic foot infection

## Abstract

*Background/Aim:* Diabetic foot infection (DFI) occurs frequently in patients, followed up with diabetic foot ulcers (DFU). For this reason, antibiotic treatment is often used in patients followed with DFU. Inappropriate use of antibiotics and increasing antibiotic resistance threaten public health. We aimed to investigate the microbial spectrum and antimicrobial resistance patterns isolated from diabetic foot infections in Turkey and help clinicians to choose optimal antibiotics empirically. *Materials and Methods*: This study was planned as a retrospective, single-center, cross-sectional study. Two hundred sixty-two patients whose causative microorganism was isolated in culture of tissue between 1 January 2021 and 31 December 2022 were included in this study. Bacterial profile and antimicrobial resistance patterns were analyzed. *Results:* Four hundred thirty two isolates from 262 patients isolated in culture of tissue were evaluated. Of these microorganisms, 57.60% were Gram-negative, 41.20% were Gram-positive bacteria, and 1.2% were *Candida* spp. The most frequently detected Gram-positive microorganism was *Staphylococcus* spp. Gram-negative microorganisms were *Escherichia coli* (*E. coli*) and *Pseudomonas aeruginosa* (*P. aeruginosa*). Polymicrobial infections were observed in 40.5% of the patients. Methicillin-resistant *Staphylococcus* spp. rate was 51.3%, while extended-spectrum beta-lactamase (ESBL) resistance for *E. coli* was 66.7%. *Conclusions:* Due to increasing antibiotic resistance rates, treatment of common infections becomes more difficult. Knowledge of the microbiological profile and antibiotic resistance patterns of patients with DFIs is useful to guide empirical therapy.

## 1. Introduction

Diabetes mellitus (DM) is an important public health problem with increasing serious complications in recent years [1,2]. According to the current data from the World Health Organization dated June 2024, there are 422 million diabetic patients in the world. The global prevalence of DM is expected to increase to 578 million people by 2030 [3]. Infections are frequently associated with the presence of DM [4]. This relationship was most evident in soft tissue infections, especially lower extremity infections [4]. Studies have shown that diabetic foot infections (DFI) carry a 50 times greater risk of hospitalization than those without DM, making it one of the most common causes of diabetes-related hospitalization [3]. Poor glycemic control, peripheral neuropathy, peripheral vascular disease, and trauma are important in the development of diabetic foot ulcer (DFU) [5]. Diabetic foot ulcer is a complication that is observed in 12–25% of patients with diabetes, and DFI develops in approximately 60% of these ulcers [1,3,6]. Increasing antibiotic resistance reduces treatment success in DFIs, as in all infections. Approximately 15% of patients followed up with DFI result in amputation. For this reason, it is much more rational to treat the underlying causes for the development of DFU. In an epidemiological study that was conducted in patients followed up with DM in Turkey, it was stated that diabetic foot wounds were seen at a rate of 14.2%, and approximately half of them had DFI [7]. This situation brings with it a serious economic burden as well as difficulties in their social lives for the patients followed up with DM. The Social Security Institution medical records are examined in Turkey; the expenditures for the treatment of diabetic foot wounds and infections are in the third place after the expenditures for cardiovascular and neurological diseases [8]. For this reason, early diagnosis and appropriate treatment are very important in order to prevent the development of osteomyelitis and amputation in patients and to minimize socioeconomic losses. For the appropriate treatment, it is necessary to bring the causative microorganism together with the appropriate antibiotic. It is known that swab cultures do not give accurate results due to the high risk of colonization and do not correlate with cultures of deep tissue taken simultaneously [9]. Tissue culture has been determined as the gold standard to eliminate the risk of colonization in the chronic wounds and to detect the correct causative agent [10]. It is reasonable to give the appropriate treatment regimen by taking cultures of tissue from all patients with open wounds. Unfortunately, we do not have the chance to obtain the culture of tissue in every center. It is known that Gram-positive bacteria are more prevalent in superficial soft tissue infections, while Gram-negative bacteria are more prevalent in deep tissue wound infections, and anaerobes should be kept in mind in necrotic wounds [11]. However, knowing the local or center-based epidemiological data allows us to follow rapidly changing antibiotic resistance surveillance. It is very important to choose the right empirical antibiotic. In this study, the patients who were followed up in the Chronic Wound Outpatient Clinic with a multidisciplinary approach within the General Surgery Clinic in our hospital due to diabetic foot were evaluated. We aimed to evaluate the cultures of tissue of the patients to define the causative microorganisms and antibiotic resistance rates of the patients, followed up with DFI. Therefore, these results may contribute to empirical antibiotic choice in DFI.

## 2. Results

**Figure 1:**
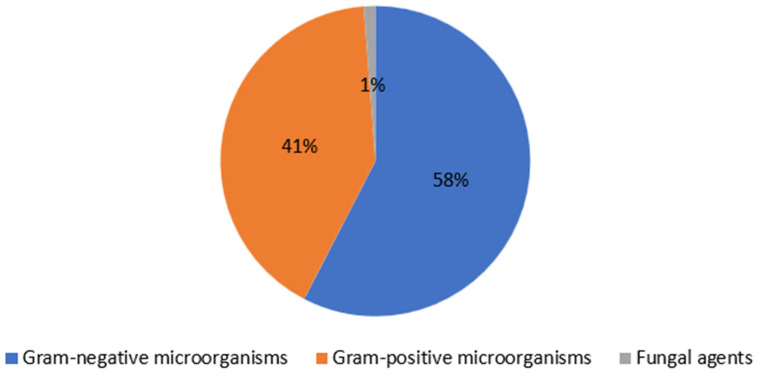
Distrubution of microorganisms isolated from cultures of tissue.

A total of 432 isolates from 262 patients were included in this study. Demographic characteristics of the patients are presented in Table 1. The mean age was 62.595 ± 11.489, and of the patients, 71.4% were male. Moreover, 16.8% of the patients had a history of amputation and 45.4% had osteomyelitis. Our study is retrospective, but the characteristics of the wounds were examined from hospital records. The wounds included in this study were moderate according to the IWGDF/IDSA 2023 classification. Of all isolates, 57.60% were Gram-negative, while 41.20% were Gram-positive bacteria, and 1.2% were *Candida* spp. (Figure 1). The most frequently detected Gram-positive microorganism was *Staphylococcus* spp., while the most frequently isolated Gram-negative microorganisms were *Escherichia coli* (*E. coli*) and *Pseudomonas aeruginosa* (*P. aeruginosa*) (Table 2). Polymicrobial infections were observed in 40.5% of the patients.

Antibiotic resistance rates of isolated Gram-positive bacteria are given in Table 3. Methicillin-resistant *Staphylococcus* spp. rate was 51.3%. Ampicillin-resistant *Enterococcus* spp. rate was 18.4%. The resistance rates of *Corynebacterium* spp. to ciprofloxacin and clindamycin were 93.5% and 54.8%, respectively (Table 3). There was no penicillin resistance in *Streptococcus* spp. Antibiotic resistance rates of isolated Gram-negative bacteria are given in Table 4. Extended-spectrum beta-lactamase (ESBL) resistance for *E. coli*, *Klebsiella* spp., and *Proteus* spp. were 66.7%, 58.6%, and 24.1%, respectively.

Microorganisms isolated from the cultures of tissues of patients with osteomyelitis were evaluated. Of the isolated microorganisms, 62.4% were Gram-negative and 37.2% were Gram-positive microorganisms. The most common Gram-negative microorganism in patients with osteomyelitis was *P. aeruginosa*, and the most common Gram-positive microorganism was *Staphylococcus* spp. (Table 5).

## 3. Materials and Methods

### 3.1. Diabetic Foot Ulcer Patients

Patients who were followed up in the Chronic Wound Outpatient Clinic of our hospital between 1 January 2021 and 31 December 2022 due to diabetic foot wounds were retrospectively analyzed. Our hospital’s Chronic Wound Outpatient Clinic is a center that works with a multidisciplinary approach. Our unit is a referral center where an average of 400 patients are evaluated per month. A total of 262 patients over 18 years of age, followed with DFI and at least one microorganism isolated in cultures of tissue, were included in this study. The patients included in this study were moderate according to IWGDF/IDSA 2023 classification [1]. Demographic characteristics of the patients (age, gender, history of amputation, presence of osteomyelitis), causative microorganisms isolated in the culture of tissue, antibiotic susceptibility results were recorded. Patients whose swab cultures were taken were not included in this study. All data were obtained from the hospital automation system. The presence of osteomyelitis was evaluated with direct radiography and magnetic resonance imaging (MRI) taken during the initial evaluation of the patient.

### 3.2. Specimen Collection

This is a retrospective study. However, we have a standard practice as mentioned when taking cultures of tissue in our chronic wound outpatient clinic. After debridement and cleaning of superficial debris with saline and 70% alcohol, a deep tissue sample is taken. After the samples are taken into a sterile container, they are kept in a 0 °C cooler until transfer. Samples are transferred to the microbiology laboratory for analysis within 30 min.

### 3.3. Microbiological Analysis

Identification of isolates in cultures of tissue was performed with a VITEK-2 (bioMérieux, Marcy-I’Ètoile, France) automated identification device. Antibiotic susceptibility tests were performed automatically on the VITEK-2 (bioMérieux, Marcy-I’Ètoile, France) identification device. Extended-spectrum beta lactamase (ESBL) production was investigated with a VITEK-2 (bioMérieux, Marcy-I’Ètoile, France) automated identification device and confirmed using a double-disk synergy test in accordance with EUCAST guidelines. Methicillin resistance was evaluated with cefoxitin. Resistance rates and MIC (minimal inhibitory concentration) values were determined according to EUCAST (the European Committee on Antimicrobial Susceptibility Testing) standards. Anaerobic bacteria were not included in this study because anaerobic cultures were not performed in our laboratory.

### 3.4. Statistical Analysis

Descriptive statistical analysis was performed using IBM SPSS Statistics 25 software (SPSS Inc., Chicago, IL, USA, 2011). Age was expressed as mean ± standard deviation. Sex, polymicrobial growth, amputation history, presence of osteomyelitis, isolated agents from tissue cultures in all patients, and patients who have osteomyelitis were expressed as numbers and percentages.

### 3.5. Ethical Approval

This study was approved by the ethics committee of Ankara Bilkent City Hospital (approval date is 9 June 2023 and approval number is E1-23-3945).

## 4. Discussion

DFI needs to be treated with a multidisciplinary approach. Surgical debridement, abscess drainage, wound care, and antimicrobial treatment should be applied together. DFI is a serious disease that can result in amputation, sepsis, and death if not treated appropriately [12]. Appropriate antibiotic therapy is one of the most important steps in DFI treatment. The ideal approach for antibiotic therapy for a patient followed up with DFI is to choose the narrowest-spectrum antibiotic with the fewest side effects, given according to the culture of tissue results. However, antibiotic treatments are usually given empirically. Empiric antibiotics are initiated early in patients who are clinically unstable or there is no chance to take a culture of tissue. For this reason, it is very important to know the local epidemiological data and the antibiotic history of the patient when choosing the appropriate antibiotic. Since some patients may also have a polymicrobial infection, it may be necessary to administer combined antibiotics to these patients. In our study, polymicrobial infection was observed in 40.5% of the patients. In a similar study, the rate of polymicrobial infection was 35.9% in DFIs [12]. Risk factors for polymicrobial infections are having a chronic deep tissue infection and previous antibiotic use [8]. However, in our study, risk factor analysis could not be performed because data on the antibiotic use history of the patients were missing.

Gram-positive microorganisms are the most common bacteria in skin and soft tissue infections [8]. In a meta-analysis, it was stated that Gram-positive microorganisms predominate in DFIs [13], while many different studies have reported Gram-negatives were the most frequent microorganisms in DFIs [11,12,14]. *S. aureus* in western countries and *P. aeruginosa* in eastern countries with hot climates are the most common microorganisms in DFIs [15,16]. The reason for this difference is not fully understood [17]. In our study, when microorganisms are evaluated, *Staphylococcus* spp. is the most common microorganism. However, when we classified the microorganisms as Gram-positive and Gram-negative microorganisms, Gram-negative microorganisms were isolated more frequently (57.60%). *E. coli* and *P. aeruginosa* are the most common Gram-negative microorganisms. In similar studies with DFI both in our country and in the world, *P. aeruginosa* and *E. coli* are among the most common Gram-negative microorganisms [12,14]. For this reason, it should be known that Gram-negative microorganisms (especially *P. aeruginosa* and *E. coli*) are the most common microorganisms when deciding on DFI empirical treatment. However, the World Health Organization also recognized antibiotic resistance as a global health threat in 2014, and treatment options are very limited due to increasing antibiotic resistance. In our study, the ESBL rate for *E. coli* reached up to 66.7%. When oral treatment options for *E. coli* are evaluated, amoxicillin-clavulanate resistance is 58.3%, ciprofloxacin resistance is 77%, and trimethoprim sulfamethoxazole resistance is 52.1%. Ciprofloxacin resistance in *P. aeruginosa is 97.9%.* Carbepenem resistance rate of *Klebsiella* spp. 13.8%, *P. aeruginosa* 35.4%, and *Acinetobacter baumannii* (*A. baumannii*) 63.6%. In our country, carbapenem resistance has been reported as 90% in *A. baumannii* strains and 50% in *Klebsiella* spp. [18]. In the point prevalence study of antibiotic use in patients hospitalized in our hospital in 2021, the rate of using at least one antibiotic was 33.8%. Carbepenems have the highest rate of inappropriate antibiotic use (17.5%) [19]. Ulcerated wounds developing in patients under follow-up due to DM are often complicated by infections [20]. For this reason, history of antibiotic use is much more frequent than in the non-diabetic population. Since our hospital is a tertiary hospital, we have many patients referred to us from different centers. These patients often have previous antibiotic use. This may cause high antibiotic resistance in patients followed up with DFI in our center.

The presence of osteomyelitis in DFIs has been noted as a risk factor for resistant microorganisms [21]. The presence of osteomyelitis being a risk factor for resistant microorganisms may also be related to frequent antibiotic use. Almost half of our cases had osteomyelitis. In similar studies conducted in our country in 2014 and 2015, the ESBL rate for *E.coli* was reported as 33%, while it was 66.7% in our study. [20,22]. This increase over the years has been alarming. Similar to Gram-negative bacteria, resistance rates were high in Gram-positive bacteria isolated in our study. MRSA is a serious problem in DFI in our country, as in many different parts of the world [21,23,24]. In a study evaluating epidemiology of DFI, methicillin resistance in *Staphylococcus* spp. was 51.1% between 2010 and 2014 in our country [24]. In our study, 63.5% of the *Staphylococcus* spp. were *Staphylococcus aureus* and 36.5% were *Coagulase-negative staphylococcus*. *Staphylococcus* spp. methicillin resistance was 51.3%. Methicillin resistance rate was 23.4% [11] for *S. aureus*. In addition, clindamycin, fucidic acid, and trimethoprim sulfomethoxazole resistances were also high. This indicates that oral treatment options for both Gram-positive and Gram-negative microorganisms are now very limited. Especially in patients followed up with osteomyelitis, the limitation of oral treatment options due to long treatment periods causes difficulties in treatment. Teicoplanin and daptomycin are outpatient parenteral treatment options for resistant Gram-positive microorganisms. In our study, Gram-negative microorganisms are more common in cases with osteomyelitis. In Turkey, outpatient parenteral treatment options for ESBL and carbapenem-resistant microorganisms are limited to ertapenem and amikacin. For this reason, prolonged hospitalization occurs in osteomyelitis caused by DFIs. Amputation occurs in patients who are not treated for an appropriate period of time.

Our study has several limitations. The first limitation is that our study is retrospective. Since it was a retrospective study, we could not have enough information about antibiotic use stories of the patients. The second limitation was that we could not identify risk factors for resistant microorganisms due to missing data. The last limitation was that anaerobic culture could not be performed. For this reason, we could not determine the role of anaerobic microorganisms in DFIs. In addition to these limitations, our study also has advantages. Our study is an epidemiological study that evaluates epidemiological data of causative agents with a large sample size. Since microbiological data are obtained from culture of tissue results, we think that we have made a correct contribution to epidemiological data. In addition, tissue cultures have been taken by the same team with the same standard technique. This minimized false results caused by incorrect collection of tissue cultures.

## 5. Conclusions

DFIs cause high morbidity and mortality. It is possible to reduce mortality and morbidity rates with early surgical and medical treatments. For this reason, a multidisciplinary treatment approach with an experienced team is required. Due to increasing antibiotic resistance rates, infection treatment becomes more difficult. Treatment should be arranged according to culture of tissue results in order to avoid unnecessary antibiotic use and to give appropriate antibiotic therapy. Data on the microbiological profile and antibiotic resistance patterns of patients with DFIs are useful for guiding the empirical therapy. For this reason, we think that our study will contribute to the epidemiological data of the country and the world.

## Figures and Tables

**Table 1:** Demographic and clinical characteristics of the patients.

Age, mean ± SD	62.595 ± 11.489
Gender (male), number (%)	187 (71.4)
Polymicrobial infections, number (%)	106 (40.5)
History of amputation, number (%)	44 (16.8)
Osteomyelitis, number (%)	119 (45.4)

**Table 2:** Microorganisms isolated from cultures of tissue, number (%).

Microorganisms	Number (%)
*Staphylococcus* spp.	74 (17.1)
*Staphylococcus aureus*	47 (10.9)
Coagulase-negative staphylococcus	27 (6.3)
*Escherichia coli*	48 (11.1)
*Pseudomonas aeruginosa*	48 (11.1)
*Enterococcus* spp.	38 (8.8)
Corynebacterium spp.	31 (7.2)
*Streptococcus* spp.	31 (7.2)
*Klebsiella* spp.	29 (6.7)
*Proteus* spp.	29 (6.7)
*Morganella morgagnii*	29 (6.7)
*Enterobacter cloacae*	16 (3.7)
*Acinetobacter baumannii*	11 (2.5)
*Serratia marcescens*	9 (2.1)
*Stenotrophomonas maltophilia*	8 (1.9)
*Candida* spp.	5 (1.2)
*Alcaligenes* spp.	5 (1.2)
*Providencia* spp.	4 (1)
*Citrobacter* spp.	4 (1)
Others	13 (3)

**Table 3:** Antibiotic resistance of isolated Gram-positive bacteria, number (%).

	*Enterococcus* spp. (n = 38)	*Staphylococcus* spp. (n = 74)	*Corynebacterium* spp. (n = 31)
Cefoxitin	-	38 (51.3)	-
Ciprofloxacin	18 (47.4)	-	29 (93.5)
Ampicillin	8 (21.1)	-	-
Amoxicillin clavulanic acid	8 (21.1)	37 (50)	-
Clindamycin	-	48 (64.9)	17 (54.8)
Tigecycline	1 (2.6)	4 (5.4)	-
Daptomycin	9 (23.7)	1 (1.4)	-
Fusidic acid	-	30 (40.5)	-
Linezolid	0 (0)	0 (0)	0 (0)
Trimethoprim sulfamethoxazole	35 (92.1)	43 (58.1)	-
Teicoplanin	3 (7.9)	0 (0)	1 (3.2)
Vancomycin	1 (2.6)	0 (0)	1 (3.2)

**Table 4:** Antibiotic resistance of isolated Gram-negative bacteria, number (%).

	ESBL	Amoxicillin Clavulanic Acid	Ciprofloxacin	Ceftriaxone	Meropenem	Piperacillin Tazobactam	Colistin	Trimethoprim Sulfamethoxazole
*Escherichia coli* (n = 48)	32 (66.7)	28 (58.3)	37 (77)	32 (66.7)	3 (6.2)	10 (20.8)	1 (2.1)	25 (52.1)
*Pseudomonas aeruginosa* (n = 48)	-	-	47 (97.9)	-	17 (35.4)	44 (91.7)	4 (8.3)	-
*Klebsiella* spp. (n = 29)	17 (58.6)	14 (48.3)	17 (58.6)	16 (55.2)	4 (13.8)	12 (41.4)	2 (6.9)	11 (37.9)
*Proteus* spp. (n = 29)	7 (24.1)	8 (27.6)	10 (34.5)	7 (24.1)	4 (13.8)	5 (17.2)	-	10 (34.5)
*Morganella morganii* (n = 29)	6 (20.7)	-	20 (69)	5 (17.2)	1 (3.4)	3 (10.3)	-	12 (41.4)
*Enterobacter cloacae* (n = 16)	4 (25)	-	2 (12.5)	4 (25)	0 (0)	1 (6.3)	1 (6.3)	0 (0)
*Acinetobacter baumannii* (n = 11)	-	-	11 (100)	-	7 (63.6)	8 (72.7)	0 (0)	7 (63.6)
*Serratia marcescens* (n = 9)	1 (11.1)	9 (100)	2 (22.2)	1 (11.1)	0 (0)	0 (0)	-	0 (0)
*Stenotrophomonas maltophilia* (n = 8)	-	-	-	-	-	-	-	0 (0)
*Alcaligenes* spp. (n = 5)	-	-	4 (80)	-	1 (20)	2 (40)	0 (0)	0 (0)
*Providencia* spp. (n = 4)	-	3 (75)	2 (50)	2 (50)	1 (25)	1 (25)	-	2 (50)
*Citrobacter* spp. (n = 4)	0 (0)	3 (75)	1 (25)	0 (0)	0 (0)	0 (0)	0 (0)	1 (25)

**Table 5:** Microorganisms isolated from patients with osteomyelitis, number (%).

Gram-negative microorganisms	166 (62.4)
Gram-positive microorganisms	99 (37.2)
Fungal agents	1 (0.3)
*Pseudomonas aeruginosa*	40 (15)
*Staphylococcus* spp.	38 (14.3)
Coagulase-negative staphylococcus	15 (5.6)
*S. aureus*	23 (8.6)
*Escherichia coli*	36 (13.5)
*Enterococcus* spp.	27 (10.2)
*Corynebacterium* spp.	22 (8.3)
*Klebsiella* spp.	20 (7.5)
*Morganella morganii*	17 (6.4)
*Proteus* spp.	12 (4.5)
*Enterobacter cloacae*	11 (4.1)
*Streptococcus* spp.	11 (4.1)
*Acinetobacter baumannii*	6 (2.3)
*Serratia marcescens*	6 (2.3)
*Alcaligenes* spp.	5 (1.9)
*Citrobacter* spp.	4 (1.5)
*Stenotrophomonas maltophilia*	4 (1.5)
*Providencia rettgeri*	2 (0.7)
*Burkholderia cepacia*	1 (0.4)
*Micrococcus luteus*	1 (0.4)
*Moraxella* spp.	1 (0.4)
*Raoultella ornithinolytica*	1 (0.4)
*Candida albicans*	1 (0.4)

## Data Availability

The data presented in this study are available upon request from the corresponding author.
